# An Accurate Calibration Method Based on Velocity in a Rotational Inertial Navigation System

**DOI:** 10.3390/s150818443

**Published:** 2015-07-28

**Authors:** Qian Zhang, Lei Wang, Zengjun Liu, Peide Feng

**Affiliations:** School of Instrument Science and Opto-Electronics Engineering, Beihang University, Beijing 100191, China; E-Mails: victoryqian175@163.com (Q.Z.); lzj0601@buaa.edu.cn (Z.L.); pdfeng@126.com (P.F.)

**Keywords:** rotational inertial navigation system (RINS), rotation modulation, rotation strategy, azimuth angle error, velocity errors

## Abstract

Rotation modulation is an effective method to enhance the accuracy of an inertial navigation system (INS) by modulating the gyroscope drifts and accelerometer bias errors into periodically varying components. The typical RINS drives the inertial measurement unit (IMU) rotation along the vertical axis and the horizontal sensors’ errors are modulated, however, the azimuth angle error is closely related to vertical gyro drift, and the vertical gyro drift also should be modulated effectively. In this paper, a new rotation strategy in a dual-axis rotational INS (RINS) is proposed and the drifts of three gyros could be modulated, respectively. Experimental results from a real dual-axis RINS demonstrate that the maximum azimuth angle error is decreased from 0.04° to less than 0.01° during 1 h. Most importantly, the changing of rotation strategy leads to some additional errors in the velocity which is unacceptable in a high-precision INS. Then the paper studies the basic reason underlying horizontal velocity errors in detail and a relevant new calibration method is designed. Experimental results show that after calibration and compensation, the fluctuation and stages in the velocity curve disappear and velocity precision is improved.

## 1. Introduction

An inertial navigation system (INS) is a fully self-contained navigation system that can continuously provide velocity, position and attitude information [1,2,3,4]. The navigation errors of an INS are mainly caused by the internal sensors, such as the gyroscope drifts and accelerometer bias [5,6]. With the development of solid-state gyroscopes, a new type of INS named rotational INS (RINS) has been proposed [7,8]. In a RINS, the inertial measurement unit (IMU) is mounted on multi-axis gimbals, and the drifts and bias errors of the internal sensors are mitigated by rotating the IMU periodically [9,10,11,12]. RINSs were implemented by NATO in the 1980s for marine inertial navigation systems and have been installed on many NATO and US warships and submarines [8,13,14]. In recent years, some new applications and improvements of RINS based on optic and MEMS sensors have been widely researched [8,9,12]. These papers illustrate how the RINS concept has been widely researched and applied, proving that the rotation scheme of the RINS has an obvious effect on navigation accuracy improvement. Theoretical analysis shows that two rotation axes should be used at least to reduce the impact of all three gyroscopes and accelerometers’ errors. Generally, the errors of horizontal sensors contribute more to the navigation system [15,16], so the typical RINS drives the IMU to rotate along the *Z* axis to modulate horizontal gyroscope drifts and accelerometer bias errors into periodically varying components. However, the vertical gyro also plays an important role in the navigation system. Supposing the drift of a vertical gyro is 0.05°/h, then the azimuth angle error will change 3′/h, which is definitely apparent and unacceptable in a high-precision INS. Several rotation strategies are presented in [17], and the simulation results show that the multi-position rotation scheme can compensate all inertial sensors’ errors. As a matter of fact, along with the complicated rotation scheme, some reasons such as whirling motion of gimbals’ axis and non-orthogonality errors of mechanical processing will give rise to the complexity of error analysis. This paper proposes a new rotation strategy that the IMU rotates bi-directionally several circles along with the *Z* axis and then quickly rotates 180° along with the *X* axis. In this way, the drifts of three gyros could be modulated according to the weight.

Due to the change of rotation strategy, the importance of calibration parameters which influence the INS is changed. The conventional calibration of INS is carried out with the support of external turntables [1,7,18,19]. In multi-axis RINS, the gimbals could be used as turntables to calibrate the error parameters of the system [5,20]. However, the accuracy of calibration parameters are all dependent on the control accuracy of gimbals in conventional calibration methods. What’s more, many error parameters are related to environmental conditions and the inertial sensors’ performance are more susceptible for environmental conditions, such as temperature [21,22], magnetic field [23], and vibration [24]. Therefore, the outline calibration methods above are not accurate enough for practical application and a precise calibration considering the system’s actual operating conditions is urgently required.

In this paper, the conventional multiposition method is treated as a basic calibration, and a backward working calibration method based on velocity error of navigation is proposed to re-calibrate some parameters precisely. The article is organized as follows: Section 2 presents the proposed RINS system configurations and mechanisms of error modulation, together with the experimental results, especially the velocity results. Section 3 analyzes the velocity errors and obtains the mathematical models between calibration parameters and velocity errors. Section 4 presents the parameter compensation algorithm and experimental results, followed by the conclusions in Section 5.

## 2. Modulation Principle and Experimental Verification

### 2.1. Modulation Principle in RINS

**Figure 1:**
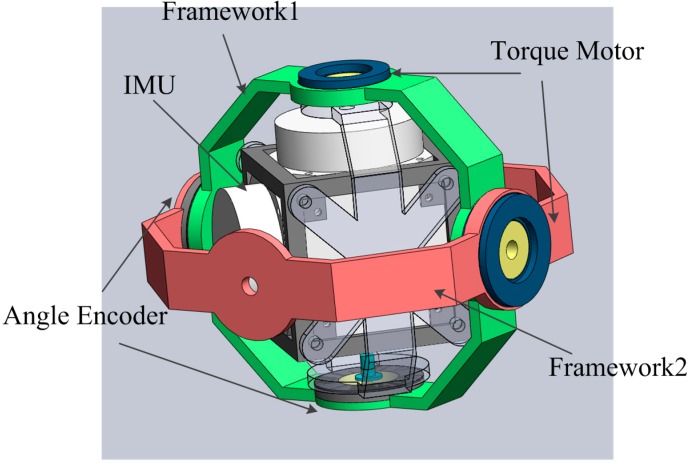
The RINS structure.

**Figure 2:**
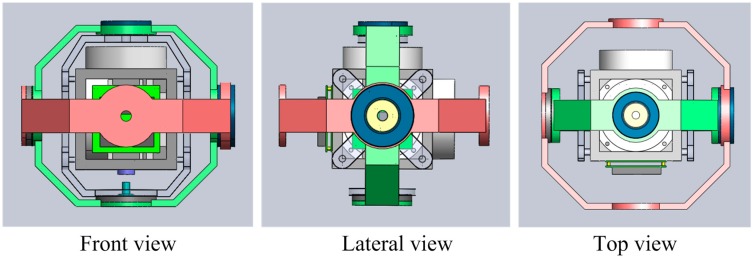
The three views of the structure.

The proposed RINS structure is shown in Figure 1 and the three views of the structure are shown in Figure 2. The RINS mainly includes two rotation frameworks, IMU, two groups of angular encoder and torque motor. The IMU could rotate continuously around the axis of Framework1, and the Framework1 including the IMU could rotate along with the axis of Framework2.

#### 2.1.1. Rotation Along with the *Z* Axis

**Figure 3:**
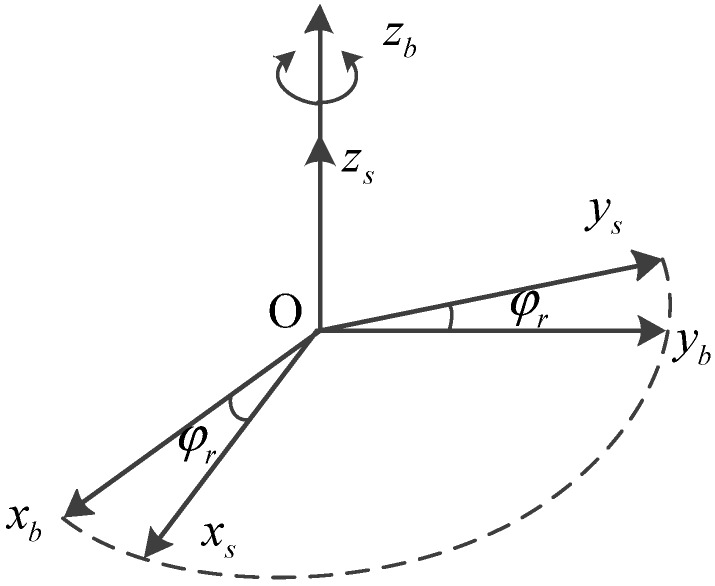
Rotation along with the *Z* axis.

The proposed system involves two essential coordinate frames. One is the inertial sensors frame (S-frame), which is varying with the changes of the IMU in real time, the other is the body frame (B-frame). As shown in Figure 3, the rotation axis $Z_{s}$ in the S-frame overlaps with the $Z_{b}$. The relationship between the two frames is defined as $\varphi_{r}$, and the S-frame coincides with the B-frame completely when the rotation angle $\varphi_{r}$ is zero.

At time *t*, the direction cosine matrix from S-frame to B-frame can be described as follows:

(1) $$C_{s}^{b}=\left[\begin{array}{ccc} {\text{cos}\omega }_{r}t & -\sin\omega_{r}t & 0\\ {\sin\omega }_{r}t & {\text{cos}\omega }_{r}t & 0\\ \text{0} & \text{0} & 1 \end{array}\right]$$

where $\omega_{r}$ is the rotation speed and $\varphi_{\text{r}}{=\omega }_{r}t$.

The geographic frame is selected as the navigation frame (N-frame). For the sake of simplicity, we just take one situation into account where the B-frame is aligned with N-frame. It means that the direction cosine matrix from B-frame to N-frame $C_{b}^{n}$ is an identity matrix. Then the angular velocity in N-frame can be described as:

(2) $$\omega^{n}=\left[\begin{array}{c} \omega_{x}^{n}\\ \omega_{y}^{n}\\ \omega_{z}^{n} \end{array}\right]=C_{b}^{n}C_{s}^{b}\omega^{s}=\left[\begin{array}{c} \omega_{x}^{s}\cos\omega_{r}t-\omega_{y}^{s}\sin\omega_{r}t\\ \omega_{x}^{s}\sin\omega_{r}t+\omega_{y}^{s}\cos\omega_{r}t\\ \omega_{z}^{s} \end{array}\right]$$

where $\omega^{n}$ and $\omega^{s}$ are the angular velocity in N-frame and S-frame, respectively.

It is obvious that the effect of rotation modulation only presents on two horizontal axes. Assuming that constant drift ${\text{ε}}_{x}^{s}$ and ${\text{ε}}_{y}^{s}$ exist in two horizontal gyros, then horizontal equivalent gyro drift in N-frame can be obtained by the following Equation:

(3) $$\left[\begin{array}{c} {\text{ε}}_{x}^{n}\\ {\text{ε}}_{x}^{n} \end{array}\right]=\left[\begin{array}{c} {\text{ε}}_{x}^{s}\cos\omega_{r}t-{\text{ε}}_{y}^{s}\sin\omega_{r}t\\ {\text{ε}}_{x}^{s}\sin\omega_{r}t+{\text{ε}}_{y}^{s}\cos\omega_{r}t \end{array}\right]$$

It can be seen that the angular velocity in B-frame is modulated from constant form into periodical form, whose average value is zero in a rotation period $T=2\text{π}/\omega_{r}$. Then the horizontal attitude errors in both rotation and strapdown mode can be calculated as follows, respectively:

(4) $$\left[\begin{array}{c} \Delta \phi_{E}\\ \Delta \phi_{N} \end{array}\right]=\left[\begin{array}{c} \int_{\;0}^{\;t}{\text{ε}}_{x}^{n}dt\\ \int_{\;0}^{\;t}{\text{ε}}_{y}^{n}dt \end{array}\right]=\left[\begin{array}{c} ({\text{ε}}_{x}^{s}\sin\omega_{r}t+{\text{ε}}_{y}^{s}\cos\omega_{r}t)/\omega_{r}-{\text{ε}}_{y}^{s}/\omega_{r}\\ (-{\text{ε}}_{x}^{s}\cos\omega_{r}t+{\text{ε}}_{y}^{s}\sin\omega_{r}t)/\omega_{r}+{\text{ε}}_{x}^{s}/\omega_{r} \end{array}\right]$$

(5) $$\left[\begin{array}{c} \Delta {\phi^{\prime }}_{E}\\ \Delta {\phi^{\prime }}_{N} \end{array}\right]=\left[\begin{array}{c} \int_{\;0}^{\;t}{\text{ε}}_{x}^{n}dt\\ \int_{\;0}^{\;t}{\text{ε}}_{y}^{n}dt \end{array}\right]=\left[\begin{array}{c} {\text{ε}}_{x}^{s}t\\ {\text{ε}}_{y}^{s}t \end{array}\right]$$

where $\Delta \phi_{*}$ is the result of rotation case, and $\Delta {\phi^{\prime }}_{*}$ is the result of strapdown case. Comparing the two equations above, it is clear that the horizontal attitude errors caused by gyro drift in rotation mode are no longer accumulated with time.

#### 2.1.2. Rotation Along with the *X* Axis

The effect of single-axis rotation modulation only presents on two horizontal sensors’ errors. In dual-axis RINS, the azimuth angle error caused by vertical gyro drift could be reduced by periodically rotating the IMU 180° with the *X* axis.

**Figure 4:**
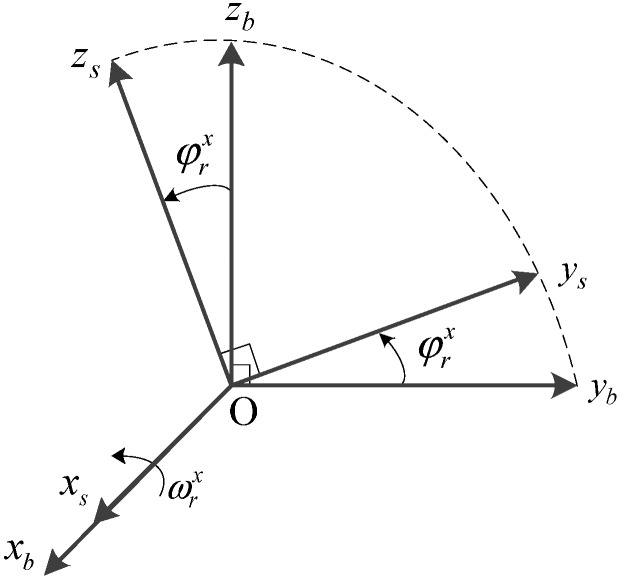
Rotation along with the *X* axis.

As shown in Figure 4, assuming that the S-frame coincides with the N-frame at the beginning, then the vertical equivalent gyro drift in the N-frame before and after rotation can be obtained by the following Equations:

(6) $$\left\{ \begin{array}{l} {\text{ε}}_{z1}^{n}={\text{ε}}_{z}^{p}={\text{ε}}_{z}\\ {\text{ε}}_{z2}^{n}=-{\text{ε}}_{z}^{p}=-{\text{ε}}_{z} \end{array}\right.$$

where ${\text{ε}}_{z1}^{n}$ and ${\text{ε}}_{z2}^{n}$ are the vertical equivalent gyro drift when the *Z* axis towards up and down, respectively. Thus the azimuth angle error engendered in this course can be obtained by integrating Equation (6) as follows:

(7) $$\Delta \phi_{U}=\int_{\;0}^{\;T}{\text{ε}}_{z1}^{n}dt+\int_{\;\text{T}}^{\;2T}{\text{ε}}_{z2}^{n}dt=\int_{\;0}^{\;T}{\text{ε}}_{z}dt+\int_{\;\text{T}}^{\;2T}-{\text{ε}}_{z}dt=0$$

Equation (7) demonstrates that the azimuth angle error could be modulated into zero by rotating the *X* axis during the time interval 2T.

### 2.2. Experimental Verification

**Figure 5:**
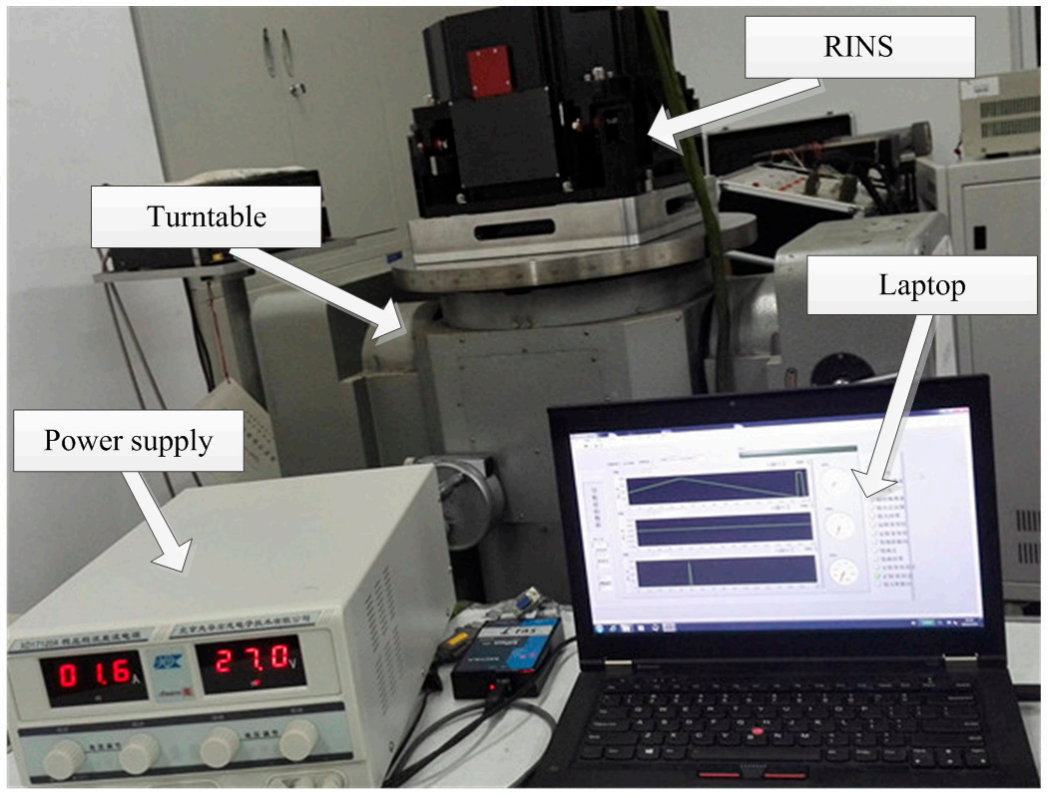
The experimental equipment.

In Figure 5, the dual-axis RINS, which has been calibrated and compensated by a conventional calibration method, is placed on a stationary marble platform. The accuracy of gyros in this experiment is 0.03°/h and the accuracy of accelerometers is 50 µg. The RINS is fed by a dc-regulated power supply, and the experimental data are collected by a laptop at a frequency of 20 Hz. The detailed rotation strategy is described as follows. At the beginning, the IMU rotates bi-directionally around the *Z* axis five times at the rotation angular speed of 6°/s, then the IMU rotates around the *X* axis 180° at the rotation angular speed of 30°/s. The modulation effect of rotation along with the *Z* axis has been analyzed comprehensively in the past [11,15], the modulation effect of azimuth angle error will be specially emphasized in the following.

**Figure 6:**
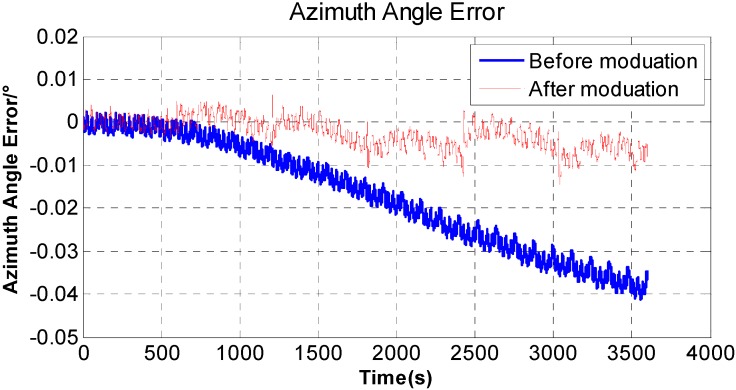
Comparison of original and modulated azimuth angle error of RINS.

In order to compare the modulation effect brought by the proposed rotation strategy, a contrastive experiment is added. The *X* axis of the system can be locked by the torque motor, and the IMU could only rotate bi-directionally along with the *Z* axis continuously. The experimental result is shown in Figure 6. The maximum of azimuth angle error with no rotation along the *X* axis is 0.04° during 1 h experiment while the maximum of azimuth angle error is only less than 0.01° in rotation mode which could reduce the impact of gyro z’s drift. Figure 6 proves that rotation along with the *X* axis do have obvious effect on restraining the azimuth angle error and improving azimuth accuracy of INS. The experimental result is consistent with Equation (7). For the change of rotation strategy, some additional errors are superimposed on the velocity curve. However, these additional errors could be compensated by calibration and algorithm which will be given in the following.

**Figure 7:**
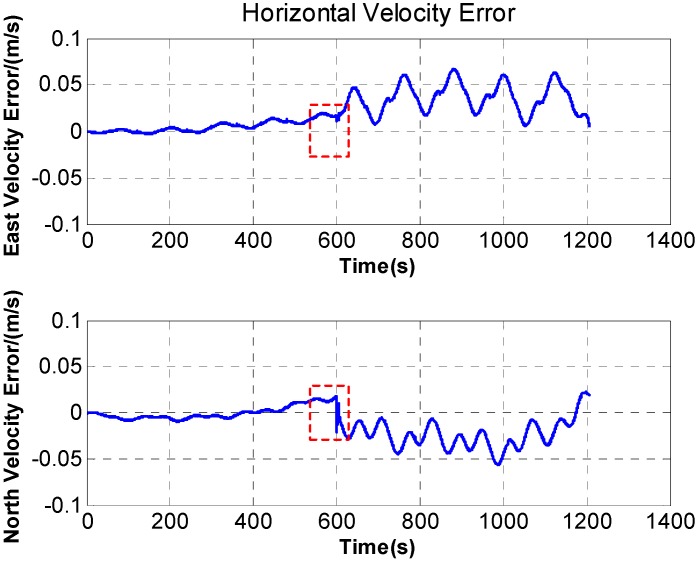
The horizontal velocity error.

**Figure 8:**
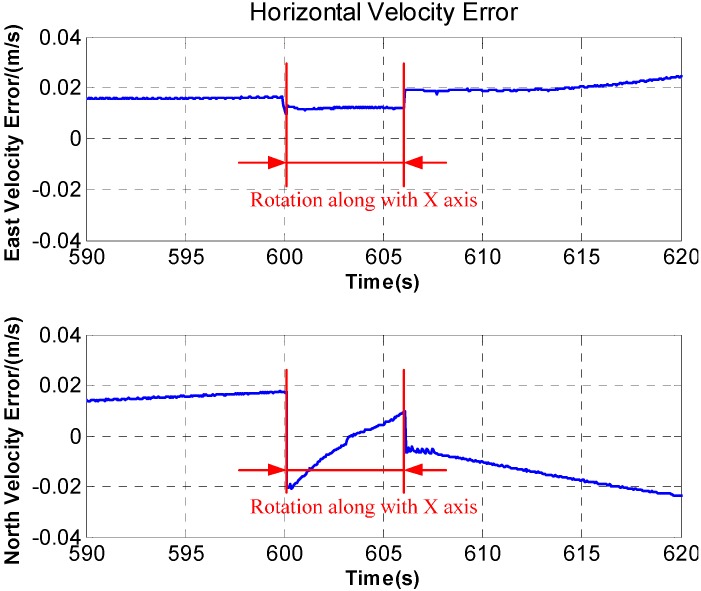
The local amplification of horizontal velocity error.

In Figure 7, after rotation along with the *X* axis, periodic fluctuation exists in the horizontal velocity error. The fluctuation is associated with rotation period, and the maximum fluctuation of the horizontal velocity error reaches 0.05 m/s. What’s more, during the rotation along with *X* axis, the velocity curve is discontinuous and a stage appears on the velocity curve. The Figure 8 is a larger version of the dotted box area in Figure 7. It is obvious that some sudden changes are present in the velocity curve at the beginning and end of the rotation.

As a conclusion of the analysis, the proposed RINS is good for azimuth accuracy. However, in the experimental result, velocity accuracy of the improved RINS is worse than that in traditional RINS mode.

## 3. Analysis of the Velocity Accuracy

### 3.1. Analysis of Velocity Fluctuation

In an inertial system, sensors’ errors and misalignment angles play a significant role in the INS error, especially for RINS [3,25]. Therefore, the S-frame defined in Section 2 is virtually a non-orthogonal coordinate frame because gyros and accelerometers are unrealistically mounted orthogonally, so the signals collected by sensors should be compensated before transforming to the B-frame.

**Figure 9:**
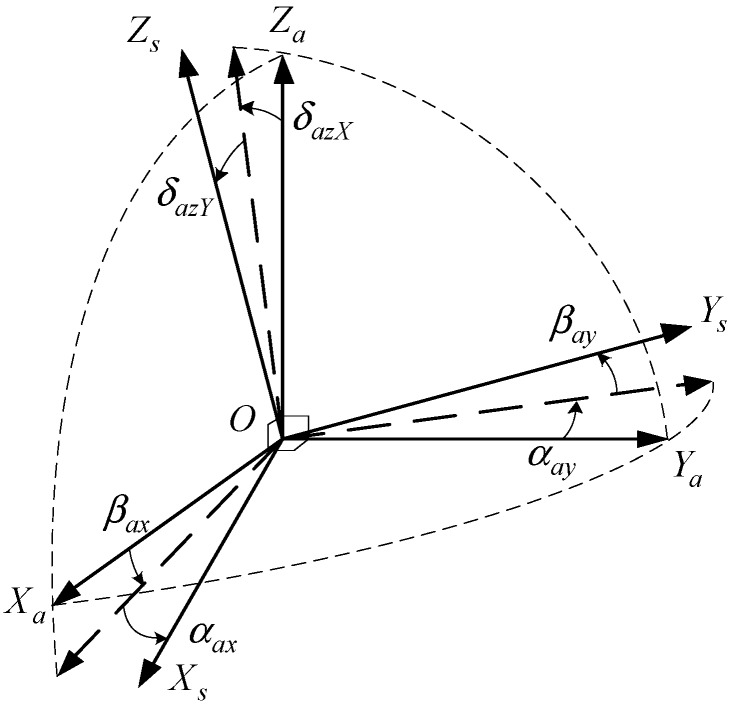
Installation errors of accelerometers.

To define the accelerometer installation errors, a new frame called A-frame is defined instead of the S-frame, and the A-frame coincides with the B-frame completely when the rotation angle ${\text{φ}}_{r}$ is zero. The relationship between S-frame and A-frame can be presented by five angles $\beta_{ax}$, $\beta_{ay}$, $\alpha_{ax}$, $\alpha_{ay}$, $\delta_{azX}$ and $\delta_{azY}$, and the detailed statement is shown in Figure 9. Then the direction cosine matrix required to transform the accelerometer data from S-frame to B-frame can be simplified as:

(8) $$C_{s}^{a}=\left[\begin{array}{ccc} 1 & -\alpha_{ax} & \beta_{ax}\\ \alpha_{ay} & 1 & -\beta_{ay}\\ \delta_{azY} & \delta_{azX} & 1 \end{array}\right]$$

where symbols $\beta_{ax}$, $\beta_{ay}$, $\alpha_{ax}$, $\alpha_{ay}$, $\delta_{azX}$ and $\delta_{azY}$ are all small angles. Accelerometer bias are symbolized as $\nabla_{x}$, $\nabla_{y}$ and $\nabla_{z}$, these nine parameters can be calibrated beforehand.

However, the accuracy of calibration of these parameters is strongly linked to the knowledge and experience of the engineer conducting the calibration process [26]. The errors of parameters will lead to extraordinary navigation error in the improved rotation strategy. The errors of installation angles are defined as $\Delta \beta_{ax}$, $\Delta \beta_{ay}$, $\Delta \alpha_{ax}$, $\Delta \alpha_{ay}$, $\Delta \delta_{azX}$ and $\Delta \delta_{azY}$, and another new coordinate frame is added to take place of previous A-frame, named real frame ($A^{\prime }$-frame). The direction cosine matrix $C_{a}^{a^{\prime }}$ can be simplified as:

(9) $$C_{a}^{a^{\prime }}=\left[\begin{array}{ccc} 1 & -\Delta \alpha_{ax} & \Delta \beta_{ax}\\ \Delta \alpha_{ay} & 1 & -\Delta \beta_{ay}\\ \Delta \delta_{azY} & \Delta \delta_{azX} & 1 \end{array}\right]$$

If the transformation relation between $A^{\prime }$-frame and A-frame can be obtained and *Z* axis points to up, the measured acceleration $f^{U}$ in $A^{\prime }$-frame can be described as:

(10) $$f^{U}=C_{a}^{a^{\prime }}C_{s}^{a}\left[\begin{array}{c} \nabla_{x}\\ \nabla_{y}\\ g+\nabla_{z} \end{array}\right]=\left[\begin{array}{c} \nabla_{x}-(\alpha_{ax}+\Delta \alpha_{ax})\nabla_{y}+(\beta_{ax}+\Delta \beta_{ax})(g+\nabla_{z})\\ \left(\alpha_{ay}+\Delta \alpha_{ay})\nabla_{x}+\nabla_{y}-(\beta_{ay}+\Delta \beta_{ay})(g+\nabla_{z}\right)\\ \left(\delta_{azY}+\Delta \delta_{azY})\nabla_{x}+(\delta_{azX}+\Delta \delta_{azX})\nabla_{y}+(g+\nabla_{z}\right) \end{array}\right]$$

where g denotes the gravity acceleration.

But in A-frame, the measured acceleration $f_{0}^{U}$ is:

(11) $$f_{0}^{U}=C_{s}^{a}\left[\begin{array}{c} \nabla_{x}\\ \nabla_{y}\\ g+\nabla_{z} \end{array}\right]=\left[\begin{array}{c} \nabla_{x}-\alpha_{ax}\nabla_{y}+\beta_{ax}(g+\nabla_{z})\\ \alpha_{ay}\nabla_{x}+\nabla_{y}-(\beta_{ay}(g+\nabla_{z})\\ \left(\delta_{azY}\nabla_{x}+\delta_{azX}\nabla_{y}+(g+\nabla_{z}\right) \end{array}\right]$$

Then, based on Equations (10) and (11), the acceleration measurement error $\Delta f^{U}$ can be described as:

(12) $$\Delta f^{U}=f^{U}-f_{0}^{U}=\left[\begin{array}{c} \Delta f_{x}^{U}\\ \Delta f_{y}^{U}\\ \Delta f_{z}^{U} \end{array}\right]$$

where:

(13) $$\left\{ \begin{array}{l} \Delta f_{x}^{U}=-\Delta \alpha_{ax}\nabla_{y}+\Delta \beta_{ax}(g+\nabla_{z})\\ \Delta f_{y}^{U}=\Delta \alpha_{ay}\nabla_{x}-\Delta \beta_{ay}(g+\nabla_{z})\\ \Delta f_{z}^{U}=\Delta \delta_{azY}\nabla_{x}+\Delta \delta_{azX}\nabla_{y} \end{array}\right.$$

As installation errors and accelerometer bias are all infinitesimal, the higher-order infinitesimal can be ignored. Then the Equation (13) can be simplified as:

(14) $$\left\{ \begin{array}{l} \Delta f_{x}^{U}=+\Delta \beta_{ax}g\\ \Delta f_{y}^{U}=-\Delta \beta_{ay}g\\ \Delta f_{z}^{U}=0 \end{array}\right.$$

Similarly, when the *Z* axis points down, the measured acceleration error $\Delta f^{D}$ can be described as:

(15) $$\left\{ \begin{array}{l} \Delta f_{x}^{D}=-\Delta \beta_{ax}g\\ \Delta f_{y}^{D}=+\Delta \beta_{ay}g\\ \Delta f_{z}^{D}=0 \end{array}\right.$$

As the acceleration measurement error $\Delta f^{U}$ and $\Delta f^{D}$ are both constant, it is coupled with the equivalent bias of accelerometer and could be calibrated and compensated as an equivalent bias. Therefore, if the equivalent bias is calibrated when the *Z* axis points to up and is compensated in the whole rotation strategy, a double equivalent bias error will exist when the *Z* axis points to down and it will be modulated into periodically varying components.

Since the measured acceleration error on the vertical axis is zero, the following analysis just focuses on two horizontal axes. According to the Equations (14) and (15), if the equivalent bias is calibrated when the *Z* axis points to up and the equivalent bias error when *Z* axis points down is described as follows:

(16) $$\left[\begin{array}{l} {\nabla^{\prime }}_{x}\\ {\nabla^{\prime }}_{y} \end{array}\right]=\left[\begin{array}{l} \Delta f_{x}^{U}-\Delta f_{x}^{D}\\ \Delta f_{y}^{U}-\Delta f_{y}^{D} \end{array}\right]=\left[\begin{array}{l} +2\Delta \beta_{ax}g\\ -2\Delta \beta_{ay}g \end{array}\right]$$

After modulation, the equivalent bias error in navigation frame can be calculated and the velocity error can also be obtained by integrating the equivalent bias error as follows:

(17) $$\left[\begin{array}{l} \nabla_{x}^{n}\\ \nabla_{y}^{n} \end{array}\right]=C_{b}^{n}C_{a}^{b}\left[\begin{array}{l} {\nabla^{\prime }}_{x}\\ {\nabla^{\prime }}_{y} \end{array}\right]=\left[\begin{array}{l} {\nabla^{\prime }}_{x}\cos\omega_{r}t+{\nabla^{\prime }}_{y}\sin\omega_{r}t\\ {\nabla^{\prime }}_{x}\sin\omega_{r}t-{\nabla^{\prime }}_{y}\cos\omega_{r}t \end{array}\right]$$

(18) $$\left[\begin{array}{l} \Delta V_{x}^{n}\\ \Delta V_{y}^{n} \end{array}\right]=\left[\begin{array}{l} \int \nabla_{x}^{n}dt\\ \int \nabla_{y}^{n}dt \end{array}\right]=\left[\begin{array}{c} \frac{{\nabla^{\prime }}_{x}}{\omega_{r}}\sin\omega_{r}t-\frac{{\nabla^{\prime }}_{y}}{\omega_{r}}\cos\omega_{r}t+d_{1}\\ -\frac{{\nabla^{\prime }}_{x}}{\omega_{r}}\cos\omega_{r}t-\frac{{\nabla^{\prime }}_{y}}{\omega_{r}}\sin\omega_{r}t+d_{2} \end{array}\right]$$

where $d_{1}$ and $d_{2}$ are all constant values.

Therefore, based on Equations (16) and (18), the velocity error can be described as:

(19) $$\left[\begin{array}{l} \Delta V_{x}^{n}\\ \Delta V_{y}^{n} \end{array}\right]=\left[\begin{array}{l} \int \nabla_{x}^{n}dt\\ \int \nabla_{y}^{n}dt \end{array}\right]=\left[\begin{array}{c} \frac{2\Delta \beta_{ax}g}{\omega_{r}}\sin\omega_{\text{r}}t+\frac{2\Delta \beta_{ay}g}{\omega_{r}}\cos\omega_{r}t+d_{1}\\ -\frac{2\Delta \beta_{ax}g}{\omega_{r}}\cos\omega_{\text{r}}t+\frac{2\Delta \beta_{ay}g}{\omega_{r}}\sin\omega_{r}t+d_{2} \end{array}\right]$$

**Figure 10:**
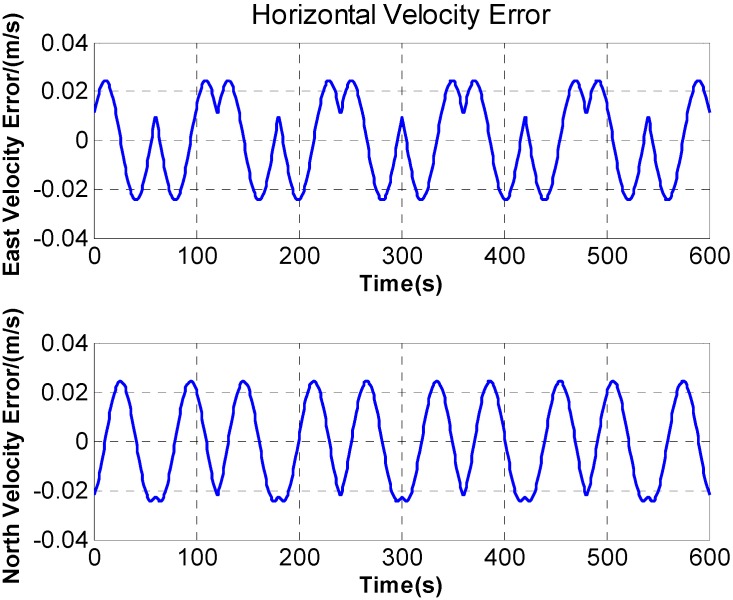
The simulation of the velocity fluctuation errors.

Figure 10 shows the simulation result in accordance with Equation (19), supposing $\omega_{r}=6{}^{\circ}/s$, $\Delta \beta_{x}=25^{\prime \prime }$, $\Delta \beta_{y}=10^{\prime \prime }$. The result shows that both horizontal velocity errors present obviously second harmonic frequency related to the rotation period of the *X* axis. It is consistent with the results in Figure 7.

### 3.2. Analysis of Velocity Stage

The horizontal gyro drifts and accelerometer bias errors are not be modulated during the rotation along with the *X* axis. In order to overcome this disadvantage, the rotation angular speed is increased to 30°/s to make the rotation last less time, which means that the motor needs a larger angular acceleration and rotation angular speed. In this way, the existing angular velocity and angular acceleration will affect the velocity error due to the lever arm [27].

**Figure 11:**
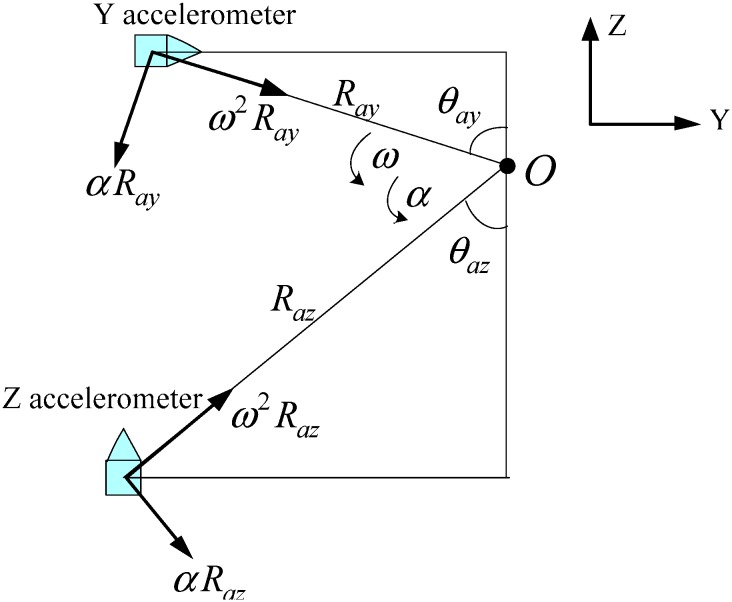
The lever arm effect curve.

The traditional lever arm effect is the measured error between the S-frame and the actual location of the sensors, which is caused by the fact the mounting position of sensors is not superposable with the rotation axis center. The direct lever arm measurement is unfaithful and is not always available when the IMU is rotating [28]. As Figure 11 shows, *O* is the rotating center, which is the *X* axis center, *R_ay_* and *R_az_* are distance values between accelerometers and rotation axis in the normal plane of the *X* axis.

As shown in Figure 11, the additional acceleration caused by the lever arm is obtained from calculation with the angular velocity and angular acceleration:

(20) $$\left[\begin{array}{l} \Delta a_{y}\\ \Delta a_{z} \end{array}\right]=\left[\begin{array}{l} \Delta a_{y1}+\Delta a_{y2}\\ \Delta a_{z1}+\Delta a_{z2} \end{array}\right]=\left[\begin{array}{l} \omega^{2}R_{ay}\sin{\text{θ}}_{ay}-\alpha R_{ay}\cos{\text{θ}}_{ay}\\ \omega^{2}R_{az}\sin{\text{θ}}_{az}-\alpha R_{az}\cos{\text{θ}}_{az} \end{array}\right]$$

where α is the angular acceleration and ω is the angular velocity.

## 4. Velocity Compensation and Experimental Results

### 4.1. Improvement in Velocity Fluctuation

Based on the analysis above, the fluctuation of velocity is caused by the coupling between the horizontal accelerometer bias and accelerometer installation errors $\Delta \beta_{ax}$ and $\Delta \beta_{ay}$. Therefore, compensating the velocity fluctuation with $\Delta \beta_{ax}$ and $\Delta \beta_{ay}$ is a fundamental method to solve the problem of velocity fluctuation error.

According to Equation (19), $\Delta \beta_{ax}$ and $\Delta \beta_{ay}$ can be obtained by fitting the velocity curve in Figure 7. However, considering the platform angle error caused by rotation, a linear function should be added in Equation (19) so that the fitting model is more consistent with the real situation. Then the compensation model can be described as follows:

(21) $$\left[\begin{array}{l} \Delta V_{x}^{n}\\ \Delta V_{y}^{n} \end{array}\right]=\left[\begin{array}{c} A_{x}\sin\omega_{r}t+B_{x}\cos\omega_{r}t+C_{x}t+D_{x}\\ A_{y}\sin\omega_{r}t+B_{y}\cos\omega_{r}t+C_{y}t+D_{y} \end{array}\right]$$

where $A_{x}=\frac{2\Delta \beta_{ax}g}{\omega_{r}}$, $B_{x}=\frac{2\Delta \beta_{ay}g}{\omega_{r}}$, $A_{y}=-\frac{2\Delta \beta_{ax}g}{\omega_{r}}$, $B_{y}=\frac{2\Delta \beta_{ay}g}{\omega_{r}}$. Clearly, $A_{x}=-A_{y}$ and $B_{x}=B_{y}$.

**Figure 12:**
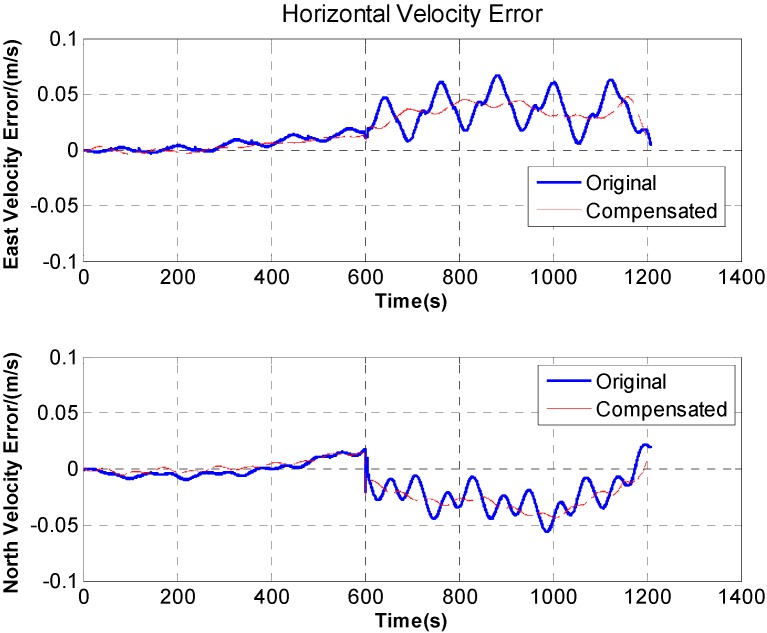
Comparison of original and compensated velocity fluctuation error.

Five experimental compensation results are listed in Table 1, and accelerometer installation errors $\Delta \beta_{ax}$ and $\Delta \beta_{ay}$ are finally calculated as −5.5″ and 12.3″. The experimental results of velocity compensation are shown in Figure 12.

**Table 1:** The coefficients of fitting.

Test Number	1	2	3	4	5
$A_{x}$	−0.0047	−0.0049	−0.0046	−0.0052	−0.0045
$B_{x}$	−0.0109	−0.0112	−0.0114	−0.0119	−0.0116
$A_{y}$	−0.0112	−0.0112	−0.0112	−0.0114	−0.0099
$B_{y}$	0.0047	0.0052	0.0051	0.0058	0.0052

Figure 12 indicates that after velocity compensation, the fluctuation on horizontal velocity almost disappeared.

### 4.2. Improvement in Velocity Stage

Figure 8 demonstrates that the velocity stage is observed at the beginning and end of the rotation along with *X* axis. Therefore, the velocity stage is mainly caused by angular acceleration. However, the middle section in Figure 8, which lasts for a long time, is mainly caused by the angular velocity because that the angular velocity is steady and the angular acceleration is zero basically. So, at the beginning and end of the rotation, the acceleration error caused by angular velocity could be ignored and angular acceleration plays a serious role, then the Equation (20) is simplified as:

(22) $$\left[\begin{array}{l} \Delta a_{y}^{1}\\ \Delta a_{z}^{1} \end{array}\right]=\left[\begin{array}{l} -\alpha R_{ay}\cos{\text{θ}}_{ay}\\ -\alpha R_{az}\cos{\text{θ}}_{az} \end{array}\right]$$

where α is the angular acceleration and could be calculated by angle encoder data.

**Figure 13:**
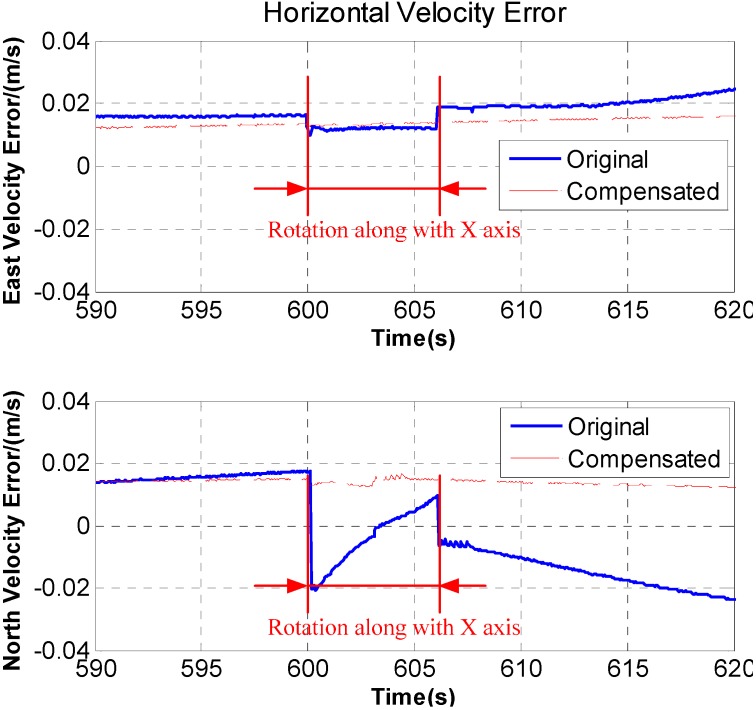
Comparison of original and compensated velocity stage error.

At the middle section in Figure 8, the angular velocity is stable at 30°/s and the angular acceleration is zero, so Equation (20) is simplified as:

(23) $$\left[\begin{array}{l} \Delta a_{y}^{2}\\ \Delta a_{z}^{2} \end{array}\right]=\left[\begin{array}{l} \omega^{2}R_{ay}\sin{\text{θ}}_{ay}\\ \omega^{2}R_{az}\sin{\text{θ}}_{az} \end{array}\right]$$

where ω=30°/s. According to Equations (22) and (23), the additional acceleration caused by the lever arm could be calculated based on the velocity stages in Figure 8. The experimental results are shown in Figure 13, it can be seen that the velocity stages disappear at the beginning and end of the rotation.

## 5. Conclusions

In this paper, a new rotation strategy is proposed to overcome the disadvantages of typical RINS that the azimuth angle error caused by azimuth gyro drift is non-convergent. The experimental results show that the proposed rotation strategy has a good inhibitory effect on azimuth angle error and the maximum of azimuth angle error is decreased from 0.04° to less than 0.01° during 1 h. However, this advanced rotation strategy leads to some additional errors on the velocity curve and it is unacceptable in a high-precision INS. Then the paper researches the basic reason behind horizontal velocity errors in detail and presented a backward working calibration method based on the velocity errors of navigation to precisely recalibrate some parameters. Experimental results show that after compensation, the fluctuation and stage in velocity curve disappears and velocity precision is improved. In addition, the proposed calibration algorithm is a general method, it is not only suitable for the proposed rotation strategy in this paper, but also it can be used to improve calibration parameters accuracy in other types of RINS with different rotation strategies. However, the experiments were all performed in a relatively stable temperature environment. While the temperature plays a role on the error parameter calibration in this paper, the effect of the temperature influence was not taken into account in this study and will further be studied as one of the future tasks.
